# Glutamate Stimulation Dysregulates AMPA Receptors-Induced Signal Transduction Pathway in Leber’s Inherited Optic Neuropathy Patient-Specific hiPSC-Derived Retinal Ganglion Cells

**DOI:** 10.3390/cells8060625

**Published:** 2019-06-21

**Authors:** Yi-Ping Yang, Phan Nguyen Nhi Nguyen, Tai-Chi Lin, Aliaksandr A. Yarmishyn, Wun-Syuan Chen, De-Kuang Hwang, Guang-Yuh Chiou, Tzu-Wei Lin, Chian-Shiu Chien, Ching-Yao Tsai, Shih-Hwa Chiou, Shih-Jen Chen, Chi-Hsien Peng, Chih-Chien Hsu

**Affiliations:** 1 Department of Medical Research, Taipei Veterans General Hospital, Taipei 112, Taiwan; molly0103@gmail.com (Y.-P.Y.); yarmishyn@gmail.com (A.A.Y.); backyard0826@gmail.com (T.-W.L.); cschien6688@gmail.com (C.-S.C.); shchiou@vghtpe.gov.tw (S.-H.C.); 2 School of Medicine, National Yang-Ming University, Taipei 112, Taiwan; taichilin@hotmail.com (T.-C.L.); m95gbk@gmail.com (D.-K.H.); dac58@tpech.gov.tw (C.-Y.T.); sjchen@vghtpe.gov.tw (S.-J.C.); 3 School of Pharmaceutical Sciences, National Yang-Ming University, Taipei 112, Taiwan; 4 Cancer Center, Taipei Veterans General Hospital, Taipei 112, Taiwan; nguyennhi204@hotmail.com; 5 Department of Neurological Surgery, Tri-Service General Hospital and National Defense Medical Center, Taipei 114, Taiwan; 6 Institute of Clinical Medicine, School of Medicine, National Yang-Ming University, Taipei 112, Taiwan; 7 Department of Ophthalmology, Taipei Veterans General Hospital, Taipei 112, Taiwan; 8 Institute of Pharmacology, National Yang-Ming University, Taipei 112, Taiwan; poohlilidada@gmail.com; 9 Department of Biological Science and Technology, College of Biological Science and Technology, National Chiao Tung University, Hsinchu 300, Taiwan; chiougy@hotmail.com; 10 Department of Ophthalmology, Taipei City Hospital, Taipei 103, Taiwan; 11 Genomic Research Center, Academia Sinica, Taipei 115, Taiwan; 12 Department of Ophthalmology, Shin Kong Wu Ho-Su Memorial Hospital, Taipei 111, Taiwan; 13 Department of Ophthalmology, Fu-Jen Catholic University, Taipei 242, Taiwan

**Keywords:** Leber’s hereditary optic neuropathy (LHON), retina, retinal ganglion cell, glutamate, AMPA receptor

## Abstract

The mitochondrial genetic disorder, Leber’s hereditary optic neuropathy (LHON), is caused by a mutation in *MT-ND4* gene, encoding NADH dehydrogenase subunit 4. It leads to the progressive death of retinal ganglion cells (RGCs) and causes visual impairment or even blindness. However, the precise mechanisms of LHON disease penetrance and progression are not completely elucidated. Human-induced pluripotent stem cells (hiPSCs) offer unique opportunities to investigate disease-relevant phenotypes and regulatory mechanisms underlying LHON pathogenesis at the cellular level. In this study, we successfully generated RGCs by differentiation of LHON patient-specific hiPSCs. We modified the protocol of differentiation to obtain a more enriched population of single-cell RGCs for LHON study. Based on assessing morphology, expression of specific markers and electrophysiological activity, we found that LHON-specific hiPSC-derived were more defective in comparison with normal wild-type RGCs. Based on our previous study, whereby by using microarray analysis we identified that the components of glutamatergic synapse signaling pathway were significantly downregulated in LHON-specific RGCs, we focused our study on glutamate-associated α-amino-3-hydroxy-5-methylisoxazole-4-propionic acid (AMPA) receptors. We found that the protein expression levels of the subunits of the AMPA receptor, GluR1 and GluR2, and their associated scaffold proteins were decreased in LHON-RGCs. By performing the co-immunoprecipitation assay, we found several differences in the efficiencies of interaction between AMPA subunits and scaffold proteins between normal and LHON-specific RGCs.

## 1. Introduction

Leber’s hereditary optic neuropathy (LHON) is a maternally inherited disorder that causes acute or subacute loss of bilateral central vision and the dysfunction of retinal ganglion cells (RGCs). The disease is characterized by incomplete penetrance and male gender bias, as it occurs in more than 50% of male LHON-associated mutation carriers and 10–15% of females [1]. The majority of LHON patients harbor one of three common mutations in the mitochondrial genome (mtDNA), namely, m.3460G>A in *MT-ND1*, m.11778G>A in *MT-ND4*, and m.14484T>C in *MT-ND6* genes. These mutations affect complex I subunits of the mitochondrial respiratory chain [2]. As a result, the adenosine-5′-triphosphate (ATP) synthesis rate is reduced, and the production and accumulation of reactive oxygen species (ROS) and oxidative stress are increased in the affected cells of LHON patients [3]. However, the underlying pathological mechanisms of LHON are still not fully understood. 

RGCs are severely affected in LHON patients [4]. Long RCG axons normally elongate to optic nerves in the brain stem and project to the visual cortex for visual information processing. RGCs constitute the only pathway through which the visual signals can integrate and transmit the information from the retina to the brain, therefore, their loss directly leads to the decrease of visual acuity and the loss of visual field. Mitochondria located in the distal axons and axonal growth cones play a crucial role during RGC development and regeneration by integrating intrinsic axon growth status with signaling from the extrinsic cues [5]. Glutamate is a major excitatory neurotransmitter of the central nervous system (CNS), which plays an important role in neurotransmission [6] and retinal development [7]. Many types of CNS-related diseases such as Parkinson’s disease, Alzheimer’s disease and Huntington’s disease, are manifested in severe neuron death due to glutamatergic excitotoxicity. Similarly to these CNS-related diseases, the death of RGCs in retinal degenerative diseases may also be caused by glutamate cytotoxicity. As was concluded from animal studies, the possible reason for the RGC death in LHON is associated with the glutamate excitotoxicity [8,9,10,11]. However, the precise mechanisms underlying LHON-related progressive RGC death and the defect of physiological functions in LHON-affected RGCs remain largely unknown.

In the mammalian CNS, the majority of fast excitatory synaptic transmission is mediated by the activity of glutamate on ionotropic/metabotropic glutamate receptors. These ionotropic glutamate receptors are tetrameric cation channels consisting of three distinct subtypes: α-amino-3-hydroxy-5-methylisoxazole-4-propionic acid (AMPA), *N*-methyl-d-aspartate (NMDA), and kainite receptors. Among them, α-amino-3-hydroxy-5-methyl-4-isoxazolepropionic acid receptors (AMPA receptors, AMPARs) conduct the majority of fast, moment-to-moment synaptic transmissions and are the primary driving force for postsynaptic depolarization [12]. AMPARs consist of tetrameric heterogenic complexes of four homologous subunits, GluR1 to GluR4, and form distinct receptor subtypes that direct different electrophysiological functions. Ca^2+^ permeability of the AMPARs is determined by their subunit composition [13]. Among the four subunits, only GluR2 (AMPA R2) is Ca^2+^-impermeable when its mRNA is specifically edited. Under the control of GluR2, it was able to protect the neuron cells from damage by excessive Ca^2+^-induced excitatory effects. Thus, the verification of the expression of each AMPAR subunit, especially the GluR2 subunit in the RGCs, was required to determine the activity and cell death on RGC.

The loss of RGCs is the primary pathological event occurring in many retinal degenerative diseases. Although currently there is no effective treatment for these diseases, cell transplantation to replace the damaged RGCs is of great potential. The human-induced pluripotent stem cells (hiPSCs) are produced by transfecting somatic cells with four stemness-associated transcription factors, and nowadays represent a powerful platform for the generation of patient-specific multilineage functional cells and tissues for autologous transplantation [14]. In addition, patient-specific hiPSCs can be differentiated to cells and tissues affected by a disease, which can be used as a disease model to investigate the molecular mechanisms of pathology and screen candidate drugs [15]. In this study, we established the in vitro disease model of LHON by differentiating LHON patient’s hiPSCs to a single type of solitary RGCs. We also investigated the mechanisms of the glutamatergic neurotransmission by AMPA receptors that were disrupted in LHON-RGCs by comparing the morphology between normal and LHON RGC cells, as well as evaluating the performance of AMPA receptors and their glutamate stimulation-induced proteins associated to AMPA receptors-related signal transduction pathway in normal and LHON RGC cells.

## 2. Materials and Methods

### 2.1. Culture of Human-Induced Pluripotent Stem Cells (hiPSCs) 

hiPSCs were cultured on Geltrex-coated dishes with mTeSR1 medium (STEMCELL Technologies, Vancouver, BC, Canada) according the manufacturer’s protocol. When the confluency reached 70–80%, hiPSCs were passaged by treatment with Accutase (Innovative Cell Technologies, San Diego, CA, USA). Cells were then maintained at 37 °C under 5% CO_2_ with 95% relative humidity.

### 2.2. Differentiation of hiPSCs to Retinal Ganglion Cells (RGCs)

The protocol of differentiation of hiPSCs to RGCs was adapted with modifications from Riazifar et al. [16]. The confluent hiPSCs were scraped off into small aggregates and transferred to non-adherent dishes containing mTeSR1 medium (STEMCELL Technologies), where they were cultivated for 10 days to generate embryoid bodies (EBs). On the tenth day, EBs were transferred to the plates coated with 0.1% gelatin and were cultivated in human embryonic stem (hES) medium supplemented with 10% fetal bovine serum (FBS), 10 mM basic fibroblast growth factor (bFGF) for eight days until neural rosettes appeared. The neural rosettes were then scraped off into suspension and cultivated for another week in hES medium supplemented with 10% FBS, 2.5 μM retinoic acid (RA) and 10 μM *N*-[*N*-(3,5-difluorophenacetyl)-l-alanyl]-*S*-phenylglycine *t*-butyl ester (DAPT) until optic vesicles were formed. The OVs were then maintained on laminin-coated dishes in DMEM/F12 containing N-2 supplement (Gibco, Carisbad, CA, USA) for one day. On the following day, the medium was replaced with Neurobasal medium/B-27 (Gibco, Carlsbad, CA, USA) supplemented with 10 μM DAPT, 50 ng/ml brain-derived neurotrophic factor (BDNF). After five days, the DAPT was removed from cultured medium. In the modified single-cell protocol, the optic vesicles (OVs) were harvested and treated with trypsin-EDTA for 5 min at 37 °C to disperse the complex structure. The single cells were passed through a 40 μm strainer and plated onto a Geltrex-coated plate for 30 min to allow the attachment of the heterogeneous population. The RGC-enriched supernatant was collected, transferred to laminin-coated plates, and was further cultivated for RGC differentiation as described above. 

### 2.3. Western Blotting

The cells were harvested and lysed in RIPA Lysis buffer (Thermo Fisher Scientific, Waltham, MA, USA) containing 1% protease inhibitor. Equal weights of total protein were separated by SDS/PAGE. After the proteins were transferred onto polyvinylidene difluoride (PVDF) membranes (Millipore, Bedford, MA, USA), the blots were incubated with blocking buffer (1 X TBST and 5% skim milk) for 1 h at room temperature and then hybridized with the primary antibodies GluR1 (1:1000; Abcam, Cambridge, UK), GluR2 (1:1000; Abcam, Cambridge, MA, USA), PICK1 (1:1000; Cell Signaling Technology, Denver, MA, USA), PKCα (1:1000; Abcam, Cambridge, MA, USA), PINK1 (1:1000; Cell Signaling Technology), Parkin (1:1000; Abcam, Cambridge, MA, USA) and Homer 1b/1c (1:700; Synaptic Systems, Goettingen, Germany) overnight at 4 °C, followed by incubation with horseradish peroxidase-conjugated secondary antibody for 1 h at room temperature. The blots were obtained by X-ray film exposure.

### 2.4. Immunoprecipitation

The Normal-RGCs and LHON-RGCs were lysed in RIPA buffer (Thermo Fisher Scientific). Protein G magnetic beads (Thermo Fisher Scientific) were washed with 0.02% Tween 20 in phosphate-buffered saline (PBS) and then incubated with the antibodies: PICK1 (5 μg; Cell Signaling Technology), Parkin (5 μg; Abcam, Cambridge, MA, USA) and GRIP1 (5 μg; Abcam, Cambridge, MA, USA) for 2 h at 4 °C. The lysate samples containing 500 μg of protein were incubated with antibody-conjugated beads in binding buffer (50 mM Hepes, 125 mM NaCl, 0.1% Triton X-100, 15 μM CaCl_2_, 5 μM EDTA) with rotation at 4 °C overnight. The beads were then washed with binding buffer four times followed by the addition of lysis buffer and sample dye. The immunoprecipitates were analyzed by immunoblotting.

### 2.5. Immunofluorescence

Cells were washed with PBS, and treated with 4% paraformaldehyde (Sigma-Aldrich, St. Louis, MO, USA) for 30 min at room temperature. After two washes with PBS, cells were permeabilized by 0.3% Triton X-100 in PBS for 10 min. After being washed with PBS four times, the cells were incubated with 3% bovine serum albumin (BSA) blocking buffer for 1 h. Primary antibodies were diluted in blocking buffer and applied onto cells for 2 h at room temperature. Primary antibodies, including BRN3a (1:200; Abcam, Cambridge, MA, USA), MAP2 (1:200, Merck Millipore, Sigma, Burlington, MA, USA), NFM (1:200; Merck Millipore), THY1 (1:500; Abcam, Cambridge, MA, USA), GluR1 (1:100; Abcam, Cambridge, MA, USA), GluR2 (1:250; Abcam, Cambridge, MA, USA), ATOH7 (1:250; Abcam, Cambridge, MA, USA), BRN3b (1:250; Santa Cruz Biotechnology, Dallas, TX, USA), F-actin (1:250; Santa Cruz Biotechnology, Dallas, TX, USA) and TuJ1 (1:500; Abcam, Cambridge, MA, USA). The cells were subsequently incubated with secondary antibodies (1:5000; Sigma-Aldrich) in blocking buffer for 1 h at room temperature. The following secondary antibodies were used: Alexa 488 goat anti-rabbit IgG, Alexa 594 goat anti-rabbit IgG, Alexa 488 goat anti-mouse IgG, and Alexa 594 goat anti-mouse IgG (Thermo Fisher Scientific). After mounting with DAPI, the slides were observed by confocal microscopy using a LSM 700 microscope (ZEISS, Jena, Germany). Immunofluorescence to track localization of the glutamate receptor unit GluR1 on the surface was carried out according to Richmond, 1996 [17].

### 2.6. Electrophysiological Analysis

The cell culture medium was replaced with artificial cerebrospinal fluid (ACSF) containing the following: 125 mM NaCl, 25 mM NaHCO_3_, 1.25 mM NaH_2_PO_4_, 2.5 mM KCl, 25 mM glucose, 2 mM CaCl_2_, and 1 mM MgCl_2_. Cell-attached and whole-cell recordings were performed with patch pipettes (3–5 MΩ) pulled from borosilicate glass tubing (outer diameter, 1.5 mm; inner diameter, 0.86 mm; Harvard Apparatus, Holliston, MA, USA) filled with internal solution containing the following: 142 mM K-gluconate, 2 mM KCl, 0.2 mM ethyleneglycol tetraacetic acid (EGTA), 4 mM MgATP, 10 mM HEPES, 7 mM Na_2_-phosphocreatine and use KOH to adjust the pH to 7.3. Signals were recorded with MultiClamp 700B amplifiers or Axopatch 200B amplifiers (Molecular Devices, Union City, CA, USA). Data were filtered at 5 kHz and sampled at 10 kHz with a Digidata 1440A interface (Molecular Devices) controlled by pCLAMP version 10.2 (Molecular Devices). The recording temperature was 22–24 °C.

### 2.7. Flow Cytometry Analysis

Differentiated RGC cells were used for phenotypic marker identification by flow cytometry. Trypsinized cells were resuspended in 100 μL of PBS and incubated with primary antibodies (anti-human CD90) (1:100 dilutions) at 4 °C for 1 h. After washing twice with PBS, labeled cells were resuspended in 100 μL of PBS with 1 μL of the fluorescein isothiocyanate (FITC)-conjugated goat anti-mouse IgG antibody (Millipore) at 4 °C for 1 h. Cells were then analyzed using a BD FACSCalibur apparatus (BD Biosciences, San Jose, CA, USA).

### 2.8. Enzyme-Linked Immunosorbent Assay (ELISA)

The concentration of glutamate secreted by Normal-RGCs and LHON-RGCs was tested by an enzyme-linked immunosorbent assay (ELISA) kit (Abcam). The conditioned medium of 7-day and 14-day RGCs was collected and the concentration of released glutamate was quantified according to the instructions provided by the manufacturer. The glutamate concentration was analyzed by the reading of OD450 on microplate spectrophotometer (Tecan, Männedorf, Switzerland).

### 2.9. Statistical Analysis

The quantifiable data are presented as the means from at least three biological replicates with standard deviation error bars. The data were compared using Student’s *t*-test, with at least *p* < 0.05 considered as statistically significant.

## 3. Results

### 3.1. Characterization of Leber’s Hereditary Optic Neuropathy (LHON) Patient 

**Figure 1:**
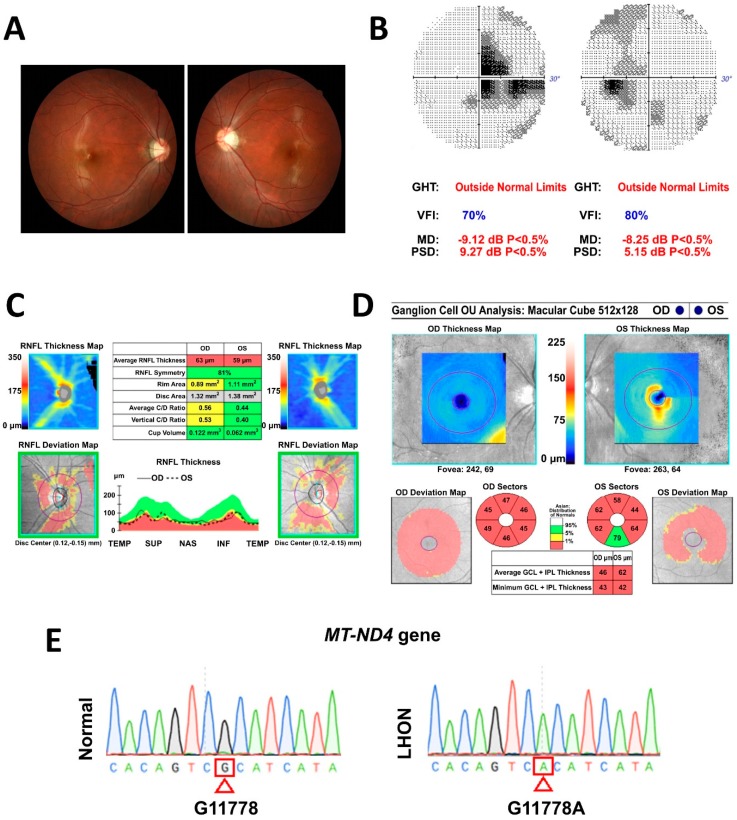
Characterization of Leber’s hereditary optic neuropathy (LHON) patient. (**A**) Fundus photography showing temporal pallor of optic disc in both eyes. (**B**) Visual filed test showing bilateral central scotoma with mean deviation of −9.12 dB in the right eye and of −8.25 dB in the left eye. (**C**) Optical coherence tomography showing decreased peripapillary average retinal fiber layer thickness of 63 μm and 59 μm in right and left eye, respectively. (**D**) Optical coherence tomography showing thinning of macular ganglion cell layers in both eyes. (**E**) DNA sequencing demonstrating the presence of G11778A mutation in patient’s mtDNA.

In this study, we aimed to generate the in vitro model of LHON by using patient-specific hiPSCs. Therefore, the cells were derived from an 18-year-old male patient presented with blurry vision for 20 months after initial presentation was diagnosed with LHON (Figure 1). On examination, the patient’s best-corrected visual acuity was 6/7.5 in the right eye and 6/10 in the left eye. Fundus photography showed temporal pallor of the bilateral optic disc (Figure 1A). Visual field testing consistently revealed bilateral central scotoma (Figure 1B). Optical coherence tomography (OCT) indicated a decrease in the peripapillary retinal nerve fiber layer (Figure 1C) and thinning of average macular ganglion cell layer in both eyes (Figure 1D). Moreover, by using sequencing, it was shown that the patient harbors *MT-ND4* G11778A point mutation of mtDNA (Figure 1E). These examinations demonstrated the loss of RGCs and axon loss of optic nerve, resulting in the reduction of visual acuity and defects in both eyes of the LHON patient.

### 3.2. Differentiation of hiPSCs to RGCs

**Figure 2:**
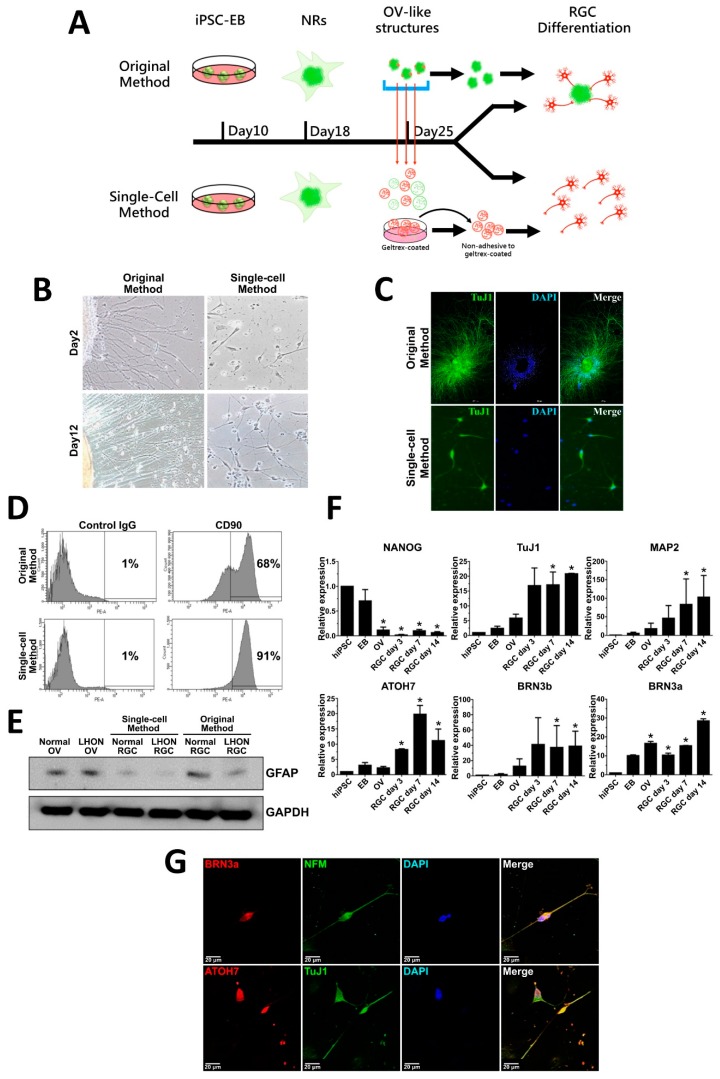
Differentiation of human-induced pluripotent stem cells (hiPSCs) from retinal ganglion cells (RGCs). (**A**) Schematic showing the timeline and procedure of differentiation of RGCs from hiPSCs by using the original and modified single-cell methods. (**B**) Bright field images of 2-day and 12-day normal hiPSC-derived RGCs obtained by the original and single-cell methods. (**C**) Immunostaining of RGC marker TuJ1 in the normal hiPSC-derived RGCs obtained by both methods. (**D**) Flow cytometry analysis of the proportion of CD90-expressing 21-day normal hiPSC-derived RGCs obtained by both methods. (**E**) Western blot showing expression of glial marker GFAP in the population of 21-day normal hiPSC-derived RGCs obtained by both methods. (**F**) qRT-PCR analysis of expression of neuronal/RGC markers and pluripotency marker NANOG in a time course of differentiation of hiPSCs to RGCs at the indicated stages. The mean fold change relative to hiPSC from three biological replicates is shown with standard deviation error bars. EB—embryoid body, NR—neural rosette, OV—optic vesicle, RGC—retinal ganglion cell, hiPSC—human-induced pluripotent stem cell. * *p* < 0.05 (Student’s *t*-test) as compared to hiPSC. (**G**) Immunostaining showing expression of RGC-specific markers BRN3a, NFM, ATOH7 and TuJ1 in 21-day normal RGCs obtained by single-cell method. Nuclei stained with DAPI. Scale bar: 20 µm.

hiPSCs were generated from peripheral blood mononuclear cells obtained from a healthy control donor and LHON patient by overexpression of four reprogramming factors, Oct-4, SOX2, c-Myc and KLF4, based on our previously published protocol [18]. RGCs were then differentiated from the generated hiPSCs in a stepwise manner following the protocol adapted from Ohlemacher [19] with several modifications as outlined in Figure 2A. First, hiPSCs were induced to form embryonic bodies (EBs) by cultivating in suspension culture. After eight days, EBs were transferred to gelatin-coated plates and grown in adherent culture for 10 days until neural rosettes (NRs) were formed. NRs were further induced to form optic vesicles (OVs) by cultivating in suspension culture for a week in the presence of certain morphogenic factors. In previous studies, hiPSC-derived RGCs were usually differentiated from OVs without separating the cell mass that composes an OV [20,21,22,23]. This cell mass includes precursors of several kinds of retinal cells, for instance, photoreceptors and glial cells, which may complicate the production of a pure neuron environment for highly specific LHON-RGC study. Hence, we modified the protocol of differentiation of OVs to RGCs in order to obtain more homogeneous population of RGCs. For this purpose, the 25-day OVs were dispersed into separate cells by trypsinization, and were passed through a strainer to separate single cells from the cell clumps. Usually, RGCs are the first cell type that appear in differentiating OVs, and these young RGCs are characterized by lower adherence than other cells in the OV cell population. Therefore, we used this differential adhesion property by briefly seeding filtered cells onto Geltrex-coated dishes, and collecting unattached young RGCs from the supernatant. These purified young RGCs were seeded onto laminin-coated dishes and were further stimulated to differentiate to mature RGCs in the course of three weeks (Figure 2A). The neurite outgrowth and axonal elongation of newly differentiated RGCs were observed from day 2 and onwards after seeding onto laminin-coated dishes (Figure 2B). Elongated neurites of RGCs obtained by the original method arose from the clumps of OV-like structures (Figure 2B, left panel), while RGCs differentiated by our modified single-cell method were clearly dispersed and separated from each other (Figure 2B, right panel). RGCs of 21 days differentiated by both methods expressed intracellular RGC marker TuJ1 indicating the maturity of the RGCs (Figure 2C). To evaluate the enrichment of RGCs isolated by our modified single-cell method, we quantified the percentage of CD90-expressing 21-day RGCs by using flow cytometry analysis (Figure 1D). Our results showed that 91% of RGCs differentiated by modified single-cell method expressed CD90 marker in contrast to 68% of CD90-positive cells in the population of RGCs obtained by unmodified original method (Figure 1D). Furthermore, by using Western blotting, we determined that the expression of glial cell marker glial fibrillary acidic protein (GFAP) was significantly lower in RGCs differentiated by the single-cell method as compared to the original method, indicating to less contamination with glial cells (Figure 2E). To assess the process of differentiation, we performed qRT-PCR to test the expression levels of mRNAs encoding a number of markers at different stages of differentiation: pluripotency marker NANOG; cytoskeleton markers TuJ1 and MAP2; specific RGC markers ATOH7, BRN3b, and BRN3a (Figure 2F). As expected, NANOG was highly expressed in hiPSCs and EBs, however, its expression dropped at the later stages of differentiation. The expression of neuronal cytoskeleton markers TuJ1 and MAP2 increased in a time-dependent manner in OVs and after RGC isolation. The mRNAs encoding ATOH7, BRB3b and BRN3a, the transcription factors important for RGC lineage specification, were also upregulated in the time course of differentiation. Using immunofluorescence, enriched 21-day RGCs were further stained for RGC-related markers BRN3a, NFM, ATOH7 and TuJ 1 to confirm their expression and distribution in single-cell RGCs (Figure 1G). As expected, BRN3a and ATOH7 were found to be predominantly expressed in the nuclei, and neuronal cytoskeleton markers NFM and TuJ1 were expressed in the neurites (Figure 1G). Taken together, our data indicated that our modified single-cell method increased the enrichment of isolated RGCs and that these single-cell RGCs exhibited morphological characteristics of RGCs and positively express RGC-related markers.

### 3.3. Enriched Single-Cell LHON-RGC Population Recapitulates LHON Pathological Features

**Figure 3:**
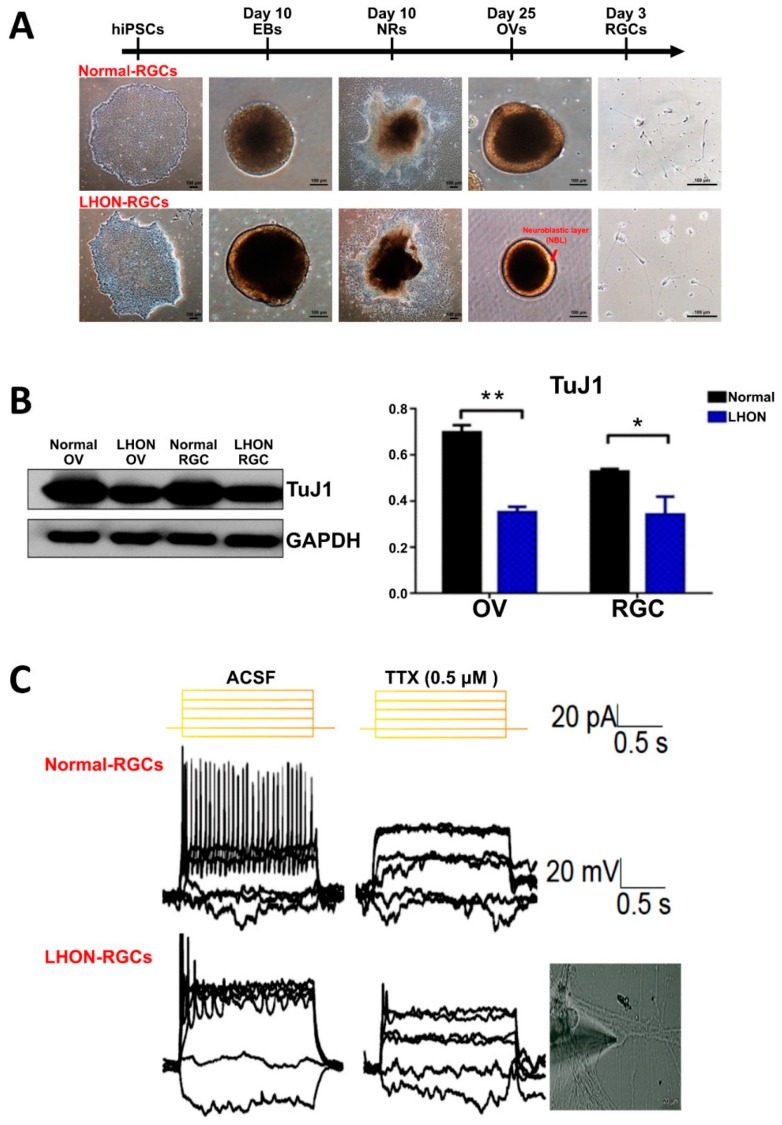
Enriched single-cell LHON-RGC population recapitulates LHON pathological features. (**A**) Bright field images showing morphologies of wild type (normal) and LHON-specific hiPSCs, EBs, NRs, OVs, and 2-day RGCs. Scale bar: 100 µm. (**B**) Western blot showing protein expression level of TuJ1 in OVs and RGCs derived from normal and LHON-specific hiPSCs. Glyceraldehyde 3-phosphate dehydrogenase (GAPDH) used as a loading control. (**C**) Electrophysiological analysis of day-21 Normal-RGCs and LHON-RGCs. Patch clamp was used to detect the spikes of action potential of cells submerged in tetrodotoxin-free artificial cerebrospinal fluid (ACSF) and ACSF containing 0.5 μM tetrodotoxin for negative control (TTX). * *p* < 0.05, ** *p* < 0.01.

Next, we investigated whether LHON-RGCs exhibit pathological features of LHON by comparing the differences between LHON-RGCs and Normal-RGCs differentiated by single-cell method. The morphologies of cells and organoids at different stages of differentiation were evaluated and no significant differences were found between normal and LHON cells at the early stages of differentiation, between hiPSCs and NRs (Figure 3A). However, at the stage of OV formation, in comparison to OVs derived from Normal-hiPSC, LHON-hiPSC-derived OVs exhibited smaller size and possessed a brighter neuroblastic layer (NBL) containing photoreceptor precursors (Figure 3A). By using the Western blot, we also found that the protein expression level of neuronal cytoskeleton marker TuJ1 was lower in both OVs and RGCs derived from LHON-hiPSCs than in the normal OVs and RGCs, respectively (Figure 3B). These findings implied that the capacity of LHON-hiPSCs to differentiate into RGCs was lower than that of Normal-hiPSCs, as was manifested in the dysfunction of dynamics of early cytoskeleton. By morphological identification, LHON-RGCs carried abnormal mitochondria which were supposed to influence deficient neuron functions. Hence, the electrophysiology of LHON-RGCs could be the key point to represent whether the ND4 mutated mitochondria would affect the neuron-signaling transduction and the maturation of neuronal ion channels. Here, we compared the electrophysiological responses of Normal-RGCs and LHON-RGCs by whole-cell patch clamp analysis (Figure 3C). The repeated firing pattern of action potential in response to the sustained current injection was noted in the 21-day Normal-RGCs. By contrast, the poor firing peaks of action potential were observed in the LHON-RGCs. Thus, the LHON-RGCs showed the dysfunction of electrophysiological activity, implying that *MT-ND4* mutation might cause defects in the ionic channel. 

### 3.4. α-Amino-3-Hydroxy-5-Methylisoxazole-4-Propionic Acid (AMPA) Receptors and AMPA Scaffold Proteins are Downregulated in LHON-RGCs

**Figure 4:**
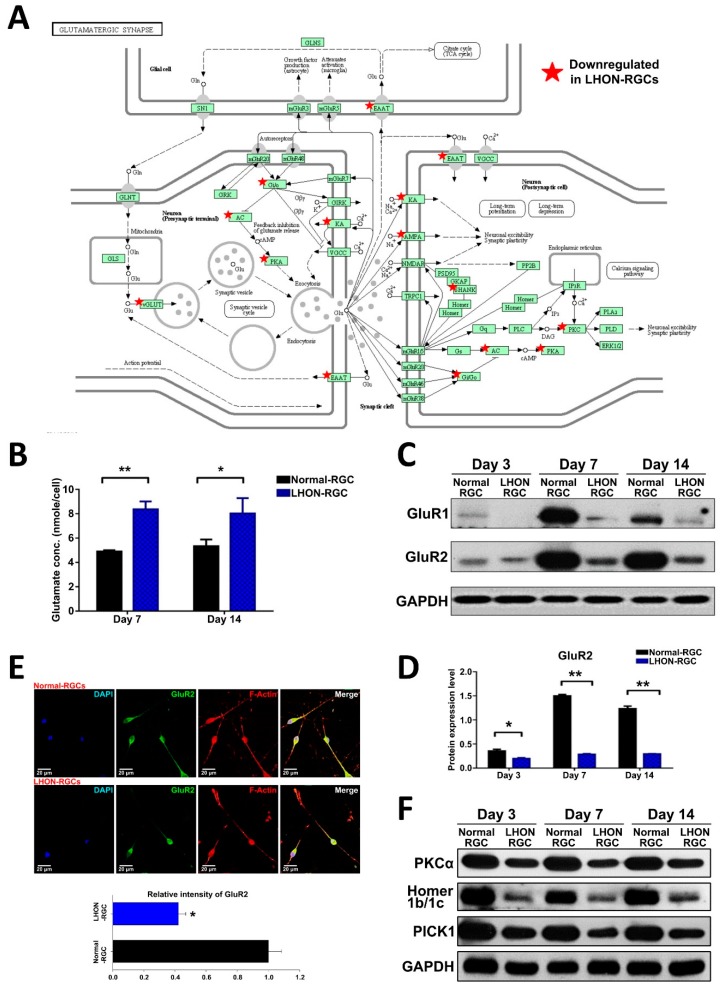
α-amino-3-hydroxy-5-methylisoxazole-4-propionic acid (AMPA) receptors and AMPA scaffold proteins are downregulated in LHON-RGCs. (**A**) Schematic showing KEGG pathway “Glutamatergic synapse” with the genes downregulated in LHON-RGCs marked with asterisks. (**B**) ELISA measurement of glutamate secreted by 7-day and 14-day Normal-RGCs and Control-RGCs. Mean concentrations from three independent measurements are shown with standard deviation error bars. (**C**) Western blot analysis demonstrating expression of GluR1 and GluR2 in day-3, day-7 and day-14 Normal-RGCs and LHON-RGCs. GAPDH used as a loading control. (**D**) Quantification of Western blot expression levels of GluR1 and GluR2. Means from three independent experiments are shown with standard deviation error bars. ** *p* < 0.01, * *p* < 0.05. (**E**) Immunofluorescent staining of GluR2 in Normal-RGCs and LHON-RGCs. Signal intensity was quantified by ImageJ and shown below. Nuclei stained with DAPI, neurites–by immunostaining of F-actin. (**F**) Western blot analysis of expression of AMPA receptor-associated scaffold proteins in Normal-RGCs and LHON-RGCs at different stages of maturity. GAPDH used as a loading control.

In our previous study, we used microarray analysis to characterize the transcriptome of LHON-RGCs [24]. By using functional enrichment analysis, we found that the genes downregulated in LHON-RGCs were most highly enriched in the Kyoto Encyclopedia of Genes and Genomes (KEGG) pathway term “Glutamatergic synapse” [24]. Among these genes were those encoding AMPA receptor components, excitatory amino acid 1 (EAAT1), various components of intracellular signaling associated with glutamatergic receptor activity (Figure 4A). The previous findings in LHON animal models demonstrated that the defective excitatory amino acid transporter 1 (EAAT1) causes glutamate accumulation in the synapses [8,9,10,11]. Taken together, these data point to the fact that glutamatergic synapse signaling is significantly affected by LHON pathology. Given the fact that AMPA receptors are the crucial components of glutamatergic synapse signaling, in this study, we sought to investigate the behavior of AMPA receptors using our hiPSC-derived LHON-RGC model. 

First, we collected the cell cultured medium at day 7 and day 14 of differentiation to measure the glutamate concentration by glutamate ELISA kit (Figure 4B). It showed that the concentration of glutamate in the LHON-RGC-conditioned medium was significantly higher than in Normal-RGC conditioned medium (Figure 4B). At the same time, by using Western blot, we showed that the protein levels of GluR1 and GluR2 were significantly decreased in LHON-RGCs compared to Normal-RGCs on days 3, 7 and 14 of differentiation (Figure 4C,D). Furthermore, we also compared the expression of GluR2 in 7-day Normal-RGCs and LHON-RGCs by immunofluorescence staining and found that the mean value of GluR2 immunofluorescence signal intensity was substantially lower in the former than in the latter (Figure 4E). GluR1 and GluR2 are known to be trafficked to the cell membrane by their associated scaffold proteins, which are crucial for GluR1/R2 activity. Therefore, by using Western blot, we showed that scaffold proteins PKCα, Homer 1b/1c and PICK1 were significantly decreased in the LHON-RGCs on days 3, 7 and 14 of differentiation, similarly to GluR1 and GluR2 (Figure 4F). To summarize, we show that AMPA receptors and the scaffold proteins crucial for their activity are highly downregulated in LHON-affected neurons.

### 3.5. Protein–Protein Interactions between AMPA Receptors and Their Associated Scaffold Proteins in LHON-RGCs

**Figure 5:**
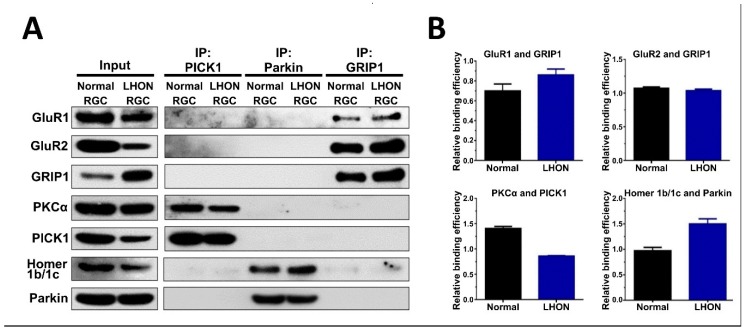
Protein–protein interactions between AMPA receptors and their associated scaffold proteins in LHON-RGCs. (**A**) Western blot analysis of PICK1, Parkin and GRIP1 immunoprecipitates from lysates of 21-day Normal-RGCs and LHON-RGCs (right panel). The aliquots of total lysates were analyzed in parallel (left panel). (**B**) Quantification of signal intensity in the indicated immunoprecipitates using ImageJ. The signal of protein binding was normalized to the signal of immunoprecipitated protein.

To investigate whether LHON-associated pathological processes interfere with interactions between AMPA receptors and their associated scaffold proteins, we performed co-immunoprecipitation (co-IP) assay in Normal-RGCs and LHON-RGCs. The lysates of these cells were subjected to immunoprecipitation using anti-PICK1, anti-Parkin and anti-GRIP1 antibodies, and by Western blot analysis of the immunoprecipitates we found that GluR1 and GluR2 associated with GRIP1, PKCα associated with PICK1, and Homer 1b/c associated with Parkin (Figure 5A). Notably, although the LHON-RGCs had poor GluR1 expression, the physical interaction between GRIP1 and GluR1 in LHON-RGCs was significantly stronger than in Normal-RGCs whereas there was no difference in the efficiency of interaction between GRIP1 and GluR2 (Figure 5B). Meanwhile, the efficiency of binding between PICK1 and PKCα in LHON-RGCs was lower than in Normal-RGCs, indicating to the reduced formation of internalized complex composed of PICK1 and PKCα (Figure 5B). However, the binding efficiency between Parkin and Homer 1b/c, the essential association for internalization, was apparently higher in LHON-RGCs, even though the expression of Homer 1b/c was decreased (Figure 5B).

### 3.6. Effects of Glutamate Stimulation on the Expression of AMPA Receptors in LHON-RGCs

**Figure 6:**
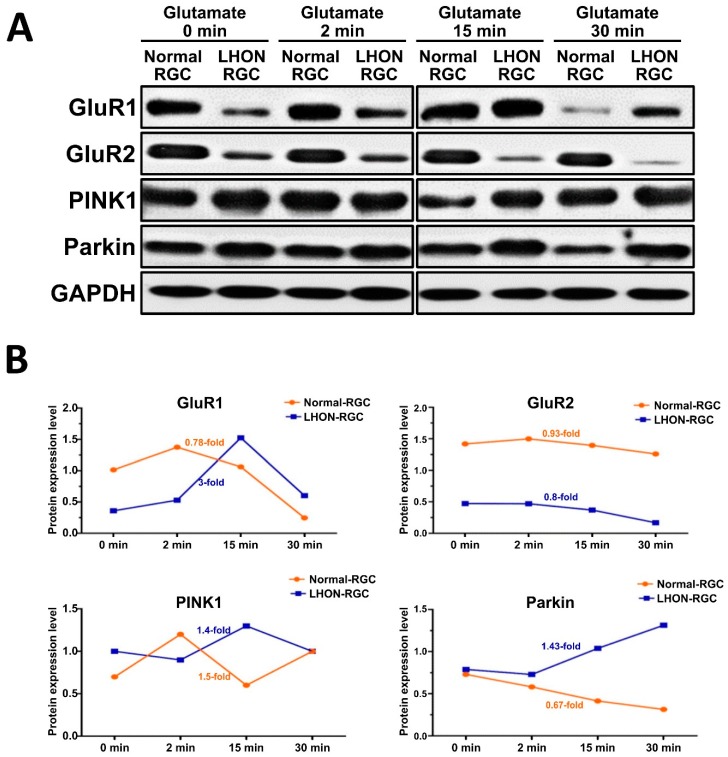
Effects of glutamate stimulation on the expression of AMPA receptors in LHON-RGCs. (**A**) Immunoblot analysis of the effect of treatment of Normal-RGCs and LHON-RGCs with 10 μM glutamate for the indicated time periods on expression levels of AMPA receptors and PINK1 and Parkin scaffold proteins. GAPDH used as a loading control (**B**) The protein expression level of GluR1/R2, PINK1 and Parkin in Normal- and LHON-RGCs were quantified by Image J.

Since we identified the differences in the expression levels and binding efficiencies between different scaffold proteins and AMPA receptors in Normal- and LHON-RGCs, next we sought to investigate the response to glutamate stimulation in these two cell lines (Figure 6). By using Western blot analysis, we evaluated the dynamics of change of protein levels of GluR1, GluR2, PINK1, and Parkin in a time course of glutamate administration (Figure 6A). Under glutamate stimulation, the expression of GluR1, GluR2, PINK1 in Normal-RGCs was slightly elevated in the first 2 min (Figure 6A,B). After 2 min, the protein expression levels declined, especially those of GluR1. In meanwhile, the expression of GluR1 and PINK1 in LHON-RGCs prominently increased after 2 min and GluR1 expression markedly declined after 15 min (Figure 6B). The expression of PINK1 in Normal-RGCs achieved its peak at 2 min before declining and rising again during 15 to 30 min. PINK1 expression in LHON-RGCs sharply increased after 2 min and stayed at high level until 30 min. Notably, the expression of Parkin showed clearly negative correlation in Normal- and LHON-RGCs. Parkin expression moderately decreased for the entire time course of stimulation in Normal-RGCs, but increased with similar rate in LHON-RGCs (Figure 6B). The elevation of Parkin and PINK1 in the first 15 min in LHON-RGCs was probably regulated by external glutamate. In contrast to what was found in LHON-RGCs, glutamate stimulation evoked the degradation of Parkin and PINK1 in Normal-RGCs (Figure 6B).

### 3.7. Trafficking of AMPA Receptors in LHON-RGCs under Glutamate Stimulation

**Figure 7:**
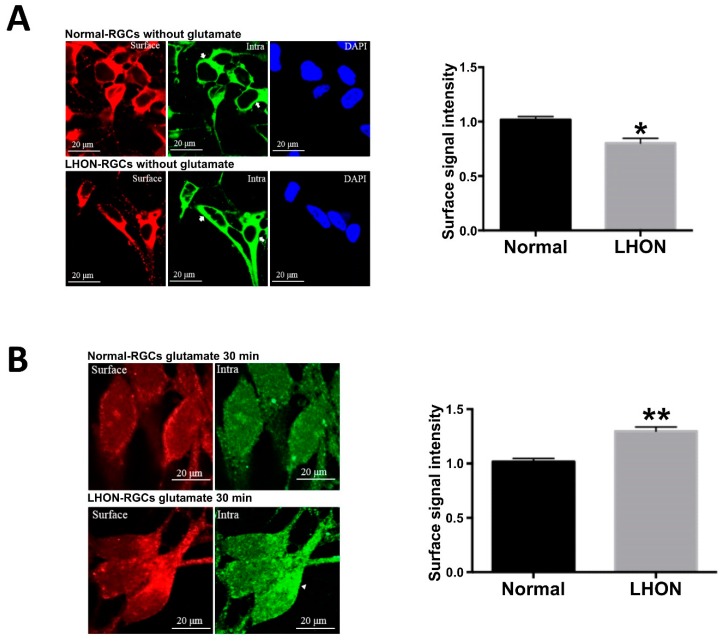
Trafficking of AMPA receptors in LHON-RGCs under glutamate stimulation. Immunofluorescent staining of surface and intracellular GluR1 in Normal-RGCs and LHON-RGCs in the absence (**A**) and presence (**B**) of glutamate stimulation. The immunofluorescence signal intensities were quantified by ImageJ (right panels). * *p* < 0.01, ** *p* < 0.001.

Since we found that the total levels of GluR1 and GluR2 vary in a time-dependent manner upon stimulation with glutamate, as the next step we sought to investigate whether the stimulation with glutamate could activate AMPA receptor internalization or trafficking in LHON-RGCs. For this purpose, we used immunofluorescent staining to identify surface and intracellular AMPA receptors in Normal-RGCs and LHON-RGCs under glutamate treatment for 30 min (Figure 7). As shown in Figure 7A, GluR1 was present in the cell membrane. The immunostaining showed that surface (red) and intracellular GluR1 (green) were located close to the cellular membrane. After glutamate treatment, GluR1 distribution was characterized by a different pattern (Figure 7B). The surface GluR1 was decreased in Normal-RGCs and LHON-RGCs after the glutamate treatment. However, the fluorescent intensity of surface GluR1 in LHON-RGCs was higher than in Normal-RGCs (Figure 7B, left panel). In addition, the intracellular signal of GluR1 was also weak in Normal-RGCs in a dispersed distribution. The green signals of intracellular GluR1 in Normal-RGCs appeared more on the inner side compared to that of LHON-RGCs. Therefore, we anticipated that the glutamate stimulation in Normal-RGCs could evoke the GluR1 internalized to intracellular location and received the accompanied degradation for the turnover. On the other hand, the glutamate might induce either GluR1 internalization or trafficking and probably caused the simultaneous localization of surfaced and intracellular GluR1.

## 4. Discussion

hiPSC technology allows us to obtain any cell type of a human organism in vitro, therefore, it offers great potential for development of in vitro disease models. In the previous studies, hiPSCs have been shown to differentiate into various retinal cell types in vitro. This promising technology accelerated and simplified the investigations of the retina development. Since RGCs appear at the early stage of retina development, it was suggested that RGCs could be isolated from the OVs at 25–30 days after the initiation of differentiation [22]. We have successfully differentiated hiPSCs derived from LHON patients and purified RGCs from the OVs [18,24]. Still, little is known about the pathogenic mechanisms of LHON. Hence, we intended to use patient-specific iPSC-based technology to investigate the mechanisms involved in LHON-RGC cells models. By understanding LHON-RGC development, we could profile the neuronal characterization and physiology and further provide the possible signs for the therapeutic treatments.

AMPA receptor trafficking and internalization mediate the variation of synaptic plasticity, especially participation in the processes of long-term potentiation (LTP) and long-term depression (LTD) [25]. Phosphorylation and ubiquitination are well-known post-translational modifications that regulate AMPA receptor trafficking and internalization. Several AMPA receptor-interacting proteins, including protein kinase Cα (PKCα), PKA and CaMKII, can regulate phosphorylation of AMPAR subunits and have been implicated in the modulation of receptor biophysical properties and receptor trafficking [12]. Ubiquitination emerged as an important regulator of AMPAR trafficking and functions. In our study, we found that binding efficiency between GluR1 and GRIP1 was significantly increased in LHON-RGCs, whereas the interaction between PICK1 and PKCα was weaker. GRIP1 is a scaffolding protein with a well-characterized role of a mediator of GluR2 anchoring to the cellular membrane. Phosphorylation of GluR2 by PKCα results in its association with the complex composed of PICK1 and PKCα, which eventually leads to GluR2 internalization. This means the GluR2 of LHON-RGCs was located on the cellular membrane occasionally with less frequency of internalization. Alteration of turnover frequency, which disrupts the functions of AMPA receptors is the leading cause of CNS neuron death in Alzheimer’s disease. It was proved that GluR2 activation of CNS neuron cells depends on the association of Homer and Parkin for its internalization. Our immunoprecipitation (IP) results indicate that the binding efficiency between Homer and Parkin is higher in LHON-RGCs than in Normal-RGCs. Therefore, we suggest to engage the IP samples to microarray analysis after the IP of Homer. 

In this study, we stimulated Normal-RGCs and LHON-RGCs with glutamate in a short-term time course to investigate the dynamics of expression of AMPA receptors and associated proteins. Upon glutamate stimulation from 2 to 15 min, the levels of GluR1, Parkin and PINK1 of Normal-RGCs declined in a time dependent manner, but their expressions in LHON-RGCs increased during this period. Only GluR2 had the same pattern of expression upon glutamate stimulation in both Normal-RGCs and LHON-RGCs. These findings point to altered responses of LHON-RGCs under glutamate stimulation. In the short-term, glutamate enhanced the expression levels of AMPAR-associated proteins. This could be due to glutamate inducing the production and trafficking of AMPA receptors to the cellular membrane as opposed to internalization. Conversely, the glutamate stimulation in Normal-RGCs could promote the internalization of the AMPA receptors for their subsequent turnover. The possible reason for the increased expression of AMPA receptors in LHON-RGCs was to compensate their original low levels in response to glutamate stimulation. In order to verify whether glutamate stimulation could influence the internalization or trafficking, we also stained the RGC cells with AMPA receptor antibodies to check the location of AMPA receptors after 30-min glutamate stimulation. The expression of surface AMPA receptors on LHON-RGCs was more obvious than that on Normal-RGCs. The intracellular intensity of AMPA receptors was also weak in Normal-RGCs. In comparison with the original expression of AMPA receptors, we proposed that glutamate stimulation could evoke their internalization in Normal-RGCs, whereas glutamate could activate the trafficking in LHON-RGCs in this duration. Therefore, for a better understanding of the dynamics of AMPA receptors under glutamate stimulation, we have to use biotin-labeled approaches to distinguish the surface and intracellular AMPA receptors in the future.

In conclusion, we first successfully generated RGCs from LHON patient-specific iPSC which showed several defects compared to those from normal RGC. The *MT-ND4*-mutated LHON-RGC cells exhibited significantly reduced GluR1/R2 and their associated scaffold proteins. The pattern of responses to glutamate stimulation in LHON-RGCs was also in contrast to that in Normal-RGCs. This study unveiled the roles of AMPA receptors’ contribution to LHON disease, and may be advantageous for developing novel therapeutic strategies.
